# Affinity of Compounds for Phosphatydylcholine-Based Immobilized Artificial Membrane—A Measure of Their Bioconcentration in Aquatic Organisms

**DOI:** 10.3390/membranes12111130

**Published:** 2022-11-11

**Authors:** Anna W. Sobańska

**Affiliations:** Department of Analytical Chemistry, Faculty of Pharmacy, Medical University of Lodz, ul. Muszyńskiego 1, 90-151 Lodz, Poland; anna.sobanska@umed.lodz.pl

**Keywords:** Immobilized artificial membrane, liquid chromatography, multiple linear regression, partial least squares regression, artificial neural networks, bioconcentration factor

## Abstract

The ***BCF*** (bioconcentration factor) of solutes in aquatic organisms is an important parameter because many undesired chemicals enter the ecosystem and affect the wildlife. Chromatographic retention factor log ***k_w_^IAM^*** obtained from immobilized artificial membrane (IAM) HPLC chromatography with buffered, aqueous mobile phases and calculated molecular descriptors obtained for a group of 120 structurally unrelated compounds were used to generate useful models of log ***BCF***. It was established that log ***k_w_^IAM^*** obtained in the conditions described in this study is not sufficient as a sole predictor of bioconcentration. Simple, potentially useful models based on log ***k_w_^IAM^*** and a selection of readily available, calculated descriptors and accounting for over 88% of total variability were generated using multiple linear regression (MLR), partial least squares (PLS) regression and artificial neural networks (ANN). The models proposed in the study were tested on an external group of 120 compounds and on a group of 40 compounds with known experimental log ***BCF*** values. It was established that a relatively simple MLR model containing four independent variables leads to satisfying ***BCF*** predictions and is more intuitive than PLS or ANN models.

## 1. Introduction

Immobilized artificial membrane (IAM) chromatography is a valuable technique used to predict the behavior of compounds towards biological membranes. IAM stationary phases based on phosphatidylcholine (PC) covalently linked to aminopropyl silica are able to mimic the natural membrane bilayer [1]. Thanks to this ability, they have become widely recognized tools for modeling drug distribution in vitro, with applications in medicinal chemistry including estimation of lipophilicity (a key feature characterizing the biological distribution of compounds), prediction of the ability of compounds to cross biological membranes (skin absorption, blood–brain barrier permeability, oral/human intestinal absorption) and estimation of other biomimetic properties (e.g., volume of distribution or Caco-2 permeability) [2,3]. More recently, immobilized artificial membrane chromatography has attracted the attention of environmental chemists, who used IAM chromatography to study the bioconcentration of pharmaceuticals [4], ecotoxicity of pesticides (expressed as LC_50_) [5] and mobility of substances in soil [6]. Applications of IAM chromatography and other phospholipid-based in vitro techniques (liposome partitioning and chromatography on unbound phosphatidylcholine stationary phases) in the studies of drug–biomembrane interactions are presented in reviews [2,3].

Anthropogenic compounds enter the aquatic environment via a number of routes, pose a threat to aquatic organisms, accumulate in their tissues and affect their fertility. The risks associated with the exposure of aquatic organisms to chemical compounds released to the environment by humans have been studied extensively, e.g., for organic sunscreens [7,8,9,10,11], per- and polyfluoroalkyl compounds [12], polycyclic aromatic hydrocarbons [13] or antibiotics [14]. There is a need to identify compounds that are potentially hazardous—bioaccumulative, persistent and toxic in the environment. The fish bioconcentration factor (***BCF***) is the ratio of the chemical concentration in the organism (***C****_B_*) and water (***C****_W_*), accounting for the absorption via the respiratory route (e.g., gills) and skin. It is commonly used to screen chemicals for their bioaccumulation potential [15], especially in the absence of the bioaccumulation factor (***BAF***),which accounts for dietary, dermal and respiratory exposures. When neither ***BAF*** nor ***BCF*** data are available, lipophilicity expressed as the octanol–water partition coefficient ***K_ow_*** is used as a surrogate measure of compounds’ ability to bioaccumulate. The criteria of bioaccumulation differ depending on regulatory agency; it is accepted that compounds that bioaccumulate have a ***BCF*** > 5000 or ***BCF*** > 2000 [16]. If no ***BCF*** or ***BAF*** data are available, it may be assumed that bioaccumulative compounds are those with log ***K_ow_*** > 5 [16,17], >4.5 [18] or >3.3 [19]. Measured and evaluated bioaccumulation data are also used to assign chemicals to three bioaccumulation categories: not significantly bioaccumulative (***BCF*** or ***BAF*** < 1000), bioaccumulative (***BCF*** or ***BAF*** between 1000 and 5000) and highly bioaccumulative (***BCF*** or ***BAF*** > 5000) [20].

Experimental toxicity data exist for just a fraction of relevant compounds, and in vivo measurements of such data require a lot of time and effort. According to Weisbrod et al., the collection of environmental toxicity data for 1240 potentially bioaccumulative compounds from the Canadian Domestic Substance List would take 82 years [15], and, as estimated in 2013, the average cost of experimental ***BCF*** determination is EUR 35,000 per compound, with more than 100 fish being sacrificed during tests lasting at least one month [21]. With the difficulties related to experimental ***BCF*** determination in mind, attention has turned to in vitro or in silico ***BCF*** models. Log ***BCF*** can be predicted using descriptors related to the partitioning of molecules between water and lipids, e.g., aqueous solubility [22,23]. However, in the majority of computational ***BCF*** models, the key descriptor governing the ability of compounds to bioconcentrate is the octanol–water partition coefficient log ***K_ow_*** (Equations (1)–(6)) [22,24,25,26,27,28].

log ***BCF*** = 0.542 log ***K_ow_*** + 0.124 (*n* = 8, R^2^ = 0.90) (1)

log ***BCF*** = 0.85 log ***K_ow_*** − 0.70 (*n* = 55, R^2^ = 0.897) (2)

log ***BCF*** = log ***K_ow_*** − 1.32 (*n* = 63, R^2^ = 0.95) (3)

log ***BCF*** = 0.94 log ***K_ow_*** − 1.00 (4)

log ***BCF*** = 0.516 log ***K_ow_*** + 0.576 (*n* = 154, R^2^ = 0.60) (5)

log ***BCF*** = 0.80 log ***K_ow_*** − 0.52 (*n* = 107, R^2^ = 0.81) (6)

It was soon noticed [29,30] that the linear log ***BCF***−log ***K_ow_*** dependencies fail for more lipophilic compounds (log ***K_ow_*** > 6 to 7), so non-linear relationships were developed (Equations (7)–(9)) [27,28]:

log ***BCF*** = −0.164 (log ***K_ow_***)^2^ + 2.069 log ***K_ow_*** − 2.592 (*n* = 154, R^2^ = 0.83) (7)

log ***BCF*** = 0.910 log ***K_ow_*** − 1.975 (6.8 ∙ 10^−7^***K_ow_*** + 1) − 0.786 (*n* = 154, R^2^ = 0.90) (8)

log ***BCF*** = 0.0069 (log ***K_ow_***)^4^ − 0.185 (log ***K_ow_***)^3^ + 1.55 (log ***K_ow_***)^2^ − 4.18 log ***K_ow_*** + 4.79 (9)

The often-observed hydrophobicity “cutoff”, i.e., the significantly reduced ability of lipophilic molecules to bioconcentrate (compared to what might be expected from their lipophilicity) is, however, disputed by some authors who attribute this phenomenon to experimental artifacts [31,32,33].

Other authors studied the influence of molecular size descriptors on the bioconcentration and bioaccumulation processes. In their opinion, molecular weight or molar volume should be incorporated in the ***BCF*** models along with log ***K_ow_*** to account for the reduced uptake of both large and highly lipophilic molecules (Equation (10) [34]):

log ***BCF*** = 3.036 log ***K_ow_*** − 0.197 (log ***K_ow_***)^2^ − 0.808 ***V****_M_* (*n* = 28, R^2^ = 0.817) (10)

where ***V_M_***—molar volume.

Dimitrov reported that the threshold value of 1.5 nm for the maximal cross-section diameter discriminates between compounds with log ***BCF***> and <3.3 [35]. Further research by Dimitrov was concerned with the influence of chemicals’ metabolism in fish liver on their ability to bioconcentrate (***BCF*** was calculated using ***K****_ow_* as the most important descriptor, with molecular size and ionization taken into account and a simulator for fish liver used to reproduce the fish metabolism) [36].

In search for models capable of addressing the hydrophobicity cutoff problem observed for highly lipophilic molecules, QSAR ***BCF*** studies were reported by several authors [18,21,37,38,39,40]. The most widely recognized models accounting for this phenomenon are:

- The model developed by Meylan [41], including different relationships depending on the compounds’ properties (Table 1);
- The model developed on the basis of Meylan’s work recommended by US EPA and available as EPI Suite^TM^ BCFBAF v. 3.02 freeware [42] (Table 1);

- CAESAR method (Equations (11)–(13)) based on eight descriptors: ***MlogP*** (Moriguchi log of the octanol–water partition coefficient), ***BEHp2*** (highest eigenvalue *n*. 2 of Burden matrix/weighted by atomic polarizabilities), ***AEige*** (absolute eigenvalue sum from electronegativity weighted distance matrix), ***GATS5v*** (Geary autocorrelation—lag 5/weighted by atomic van der Waals volumes), ***Cl-089*** (Cl attached to C1(sp^2^)), X0sol (solvation connectivity index chi-0), ***MATS5v*** (Moran autocorrelation—lag 5/weighted by atomic van der Waals volumes), ***SsCl*** (sum of all (–Cl) E-state values in molecule) [37,43]. According to the CAESAR method, ***BCF*** is calculated according to two models, A and B, whichdiffer in the selection of descriptors (with ***MlogP*** and ***BEHp2*** being common to A and B), and the *BCF* value is finally predicted as follows:

log ***K_ow_*** ≤ 1.355   log ***BCF*** = 0.936 log ***BCF_mean_*** − 0.123 (11)

1.355 ≤ log ***K_ow_*** ≤ 2.410 log ***BCF*** = 0.996 min(log ***BCF_A_***, log ***BCF_B_***) (12)

log ***K_ow_*** > 2.410   log ***BCF*** = 1.052 log ***BCF_mean_*** (13)

- The model suggested by the Technical Guidance Document (TGD) on risk assessment [18] (Equations (14)–(17)):

log ***K_ow_*** < 1   log ***BCF*** = 0.15 (14)

1 ≤ log ***K_ow_*** ≤ 6   log ***BCF*** = 0.85 log ***K_ow_*** − 0.70 (15)

6 < log ***K_ow_*** < 10 log ***BCF*** = −0.20 (log ***K_ow_***)^2^ + 2.74 log ***K_ow_*** − 4.72 (16)

log ***K_ow_*** ≥ 10   log ***BCF*** = 2.68 (17)

Alternative approaches to ***BCF*** predictions involve molecular connectivity indices [44,45,46] or solvation parameters [47]. ***BCF*** may also be estimated on the basis of quantum chemical descriptors [48] (Equation (18)):

***BCF*** = 0.00250 ***M*_w_** − 0.0724 ***E_T_*** − 0.214 ***E_HOMO_*** − 0.892 ***E_LUMO_*** − 2.58 (18)

where ***E_T_***—total energy (hartree), ***E_HOMO_***—energy of the highest occupied molecular orbital (eV) and ***E_LUMO_***—energy of the lowest unoccupied molecular orbital (eV).

The ***BCF*** can be estimated using reversed-phase chromatographic retention data. In particular, retention parameters derived from HPLC chromatography on C_18_, C_8_, C_2_ and phenyl-bonded silica sorbents were used as predictors of ***BCF*** of aromatic hydrocarbons [49]. C_18_ and cyanopropyl- and phenyl-bonded silica were used in chromatographic bioconcentration studies of aromatic hydrocarbons, alkylbenzenes, chlorinated benzenes, phthalates, nitroaromatics, phenols and aniline [50]. RP-18 TLC chromatographic descriptors were used to investigate the bioconcentration factors of organic sunscreens and cosmetic preservatives [51]. IAM chromatographic descriptors were used to study the ***BCF*** of structurally unrelated chemicals [4].

The objective of this study was to develop useful and easy-to-use predictive models of the bioconcentration factor of structurally diverse solutes based on their affinity for phosphatydylocholine-based artificial membranes. Novel models proposed in this study were generated using multiple linear regression (MLR), partial least squares (PLS) and artificial neural network (ANN) techniques. It is the first report on PLS and ANN approaches to bioconcentration studies involving chromatographic and calculated physico-chemical data.

## 2. Materials and Methods

### 2.1. Compounds, IAM Chromatographic Data, Reference BCF Values

The first stage of this study was intended to involve 175 compounds, whose IAM chromatographic retention factors obtained for purely aqueous mobile phases (log ***k_w_***^IAM^) were compiled by Sprunger et al. [52]. Because of the lack of experimental ***BCF*** data for the whole group of 175 compounds, log ***BCF*** (denoted later as log ***BCF_EPI_***) was calculated using the commonly accepted computational approach (EPI Suite^TM^, BCFBAF module v. 3.02) [42] based on Meylan’s model [41]. A large number of compounds considered at this stage of the study, however, were molecules with arbitrarily assigned log ***BCF*** = 0.50. The majority of such compounds were excluded from the training set because it was suspected that their theoretical log ***BCF*** value may not truly reflect their ability to bioconcentrate [4], and the models generated in this study were finally based upon a solute set containing 120 compounds from different chemical families (***1*** to ***120***). The excluded compounds were later combined with solutes, whose log ***k_w_***^IAM^ values were reported by other authors [53,54], to form an external test set also containing 120 compounds (***121*** to ***240***) with and without known experimental values of log ***BCF*** (log ***BCF_vivo_***). Reliable reference log ***BCF*** values (log ***BCF_EPI_***) were available for compounds ***1*** to ***187,*** and, for the compounds ***188*** to ***240***, log ***BCF*** was calculated de novo. The external set of compounds included more lipophilic molecules, whose log ***k_w_***^IAM^ could not be measured directly by chromatography with 100% aqueous mobile phase and could only be calculated by extrapolation of log ***k***^IAM^ vs. the *φ* plots obtained for a series of chromatographic experiments with mobile phases containing different concentrations *φ* of a water-miscible organic solvent, usually according to the linear Soczewiński–Wachmeister Equation (19) [55]:

log ***k*** = log ***k****_w_* + *S****φ*** (19)

The values of log ***BCF_vivo_*** were taken from the literature sources and the EPISuite^TM^ database [4,29,36,42]. The reference log ***BCF_EPI_*** and the experimental log ***BCF_vivo_*** values for compounds ***1*** to ***240*** (where available) are given in Table S1 (Supplementary Materials); the IAM chromatographic retention factors are given in Table S2 (Supplementary Materials).

### 2.2. Calculated Descriptors

Molecular weight (***M_w_***), heavy atom count (***#HvAt***), aromatic heavy atom count (***#ArHvAt***), fraction of sp^3^ carbons (***F_Csp3_***), rotable bond count (***FRB***), hydrogen donor count (***HD***), hydrogen acceptor count (***HA***), molecular refractivity (***MR***) and topological polar surface area (***TPSA***) were calculated using SwissADME software available freely on-line [56]. Total energy (***E_t_***), energy of the highest occupied molecular orbital (***E_HOMO_***), energy of the lowest unoccupied molecular orbital (***E_LUMO_***), dipole point charge (***DiPCh***), dipole hybrid (***DipH***) and dipole sum (***DipS***) were of Mopac 2016 type and were calculated using the OCHEM platform [57]. Octanol–water partition coefficient (log ***K_ow_***) was calculated according to the KOWWIN algorithm [58] using EPI Suite^TM^ software [42] (Table S2).

### 2.3. Partial Least Squares Approach

Multiple linear regression (MLR) is a common approach used in QSAR studies.It is based on the assumption that the effect of a set of molecule’s properties on its activity is additive, and the properties are (almost) independent. The conditions that must be satisfied to generate reliable MLR models are severe—standard regression techniques based on the least squares estimation give unstable and unreliable results when independent variables are colinear, and the number of cases must exceed the number of variables (ideally, it should be at least five times greater). In order to overcome the colinearity problem, partial least square (PLS) regression was developed. PLS replaces the original variables with “components”—linear combinations of the variables based on the correlation between the dependent variable and the independent variable(s) [59,60].

Regression models based on PLS estimation must be optimized in terms of the number of components—if too many are used, a model is over-fitted (it perfectly fits the training dataset, but it gives poor prediction results for new cases); if too few components are used, the model is under-fitted (it is not sufficiently large to capture the important data variability). Models based on the same number of components can be compared using RSS (residual sum of squares) or R^2^, but these parameters are unsuitable for models with different numbers of components. PLS models are often evaluated using RMSEP calculated for a separate test set and/or using cross-validation—RMSEP usually decreases as more variables are added to a small model, then it stabilizes around the optimum number of components, and it increases when the model becomes over-fitted [61].

### 2.4. Statistical Tools

Multiple linear regression (MLR) models were generated using Statistica v. 13 by StatSoft Polska, Kraków, Poland, stepwise forward regression mode. Partial least squares (PLS) models were generated using Statistica v. 13, NIPALS algorithm with auto-scaling. Multilayer Perceptron(MLP) artificial neural networks (ANNs), with the number of inputs the same as the number of variables, the varying number of hidden units and one output unit, were generated using Statistica v. 13 (regression mode, Automated Network Search—ANS module, 1000 networks to train, 50 networks to retain). The neuron activation functions were selected from the following group: identity, logistic, hyperbolic tangent and exponential. The BFGS (Broyden–Fletcher–Goldfarb–Shanno) algorithm was used to train the network together with the sum of squares (SOS) error function.

The models considered in this study were evaluated using the following procedures and statistical parameters:

- K-fold cross-validation, with *n* compounds from the initial training set split into *k* even subsets, (*k* − 1) of which were used to train a new model and the remaining one to test it; the procedure was repeated *k* times, each time using a different subset of compounds as a test set. After each cross-validation step, the RMSE (root mean squared error) was calculated for the particular N-compound test subset according to the following Equation (20):

(20) $$\mathrm{RMSE}=\sqrt{\frac{\sum_{i=1}^{N}{\left(y_{i}^{pred}-y_{i}^{ref}\right)}^{2}}{N}}$$

The overall root mean squared error of k-fold cross-validation (RMSECV) is calculated as follows:

(21) $$\mathrm{RMSECV}=\sqrt{\frac{\sum_{j=1}^{k}{\mathrm{RMSE}}_{j}^{2}}{k}}$$

In this study, *n* = 120, *k* = 5 and N = 24; *y_i_^pred^* and *y_i_^ref^* are log ***BCF_pred_*** and log ***BCF_EPI_***, respectively.

- Relationship between the predicted log ***BCF_pred_*** values (computed for the external test set of 67 compounds ***121*** to ***187*** that were not used to build models) with the reference values log ***BCF_EPI_***—using root mean squared error of prediction (RMSEP_ext_), calculated according to Equation (20);
- Comparison of the predicted log ***BCF_pred_*** values (calculated for 40 compounds, whose experimental log ***BCF_vivo_*** data are available), and these data—using squared coefficient of determination (R^2^_vivo_) and root-mean-squared error of prediction (RMSEP_vivo_), calculated according to Equation (20).

## 3. Results and Discussion

### 3.1. Multiple Linear Regression (MLR) Models

**Figure 1:**
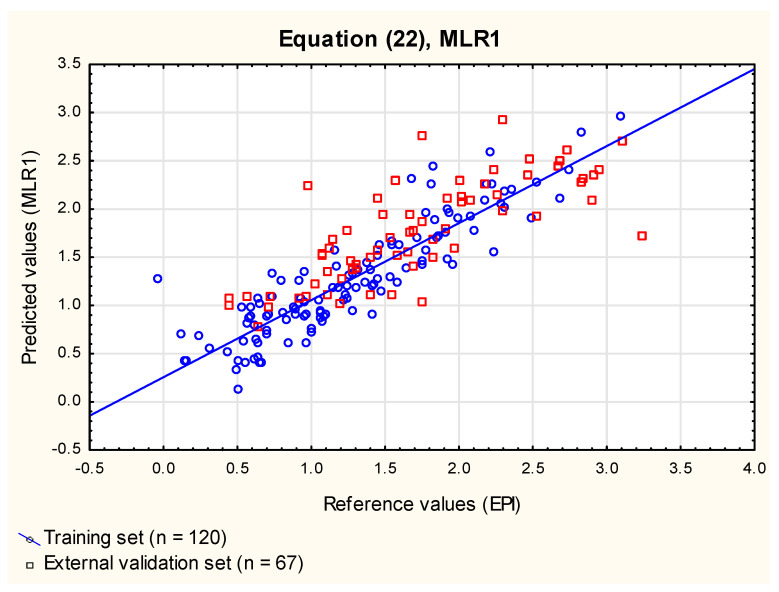
MLR1 model, Equation (22)—predicted vs. reference log BCF values.

The values of log ***BCF_EPI_***, calculated for compounds ***1*** to ***120*** using EPISuite^TM^ software [42], were plotted against the IAM retention factors obtained for aqueous mobile phases log ***k_w_***^IAM^ and compiled by Sprunger [52]. The linear relationship between log ***BCF_EPI_*** and log ***k_w_***^IAM^ (Equation (22), **MLR1**, Figure 1) accounted for 80% of total log ***BCF_EPI_*** variability.

log ***BCF***_EPI_ = 0.18 (±0.06) + 0.70 (±0.03) log ***k_w_***^IAM^ (22)

(*n* = 120, R^2^ = 0.80, R^2^_adj_ = 0.80, RMSECV = 0.30, RMSEP_ext_ = 0.45, RMSEP_vivo_ = 0.35, R^2^_vivo_ = 0.74, F = 470.5, *p* < 0.01).

**Figure 2:**
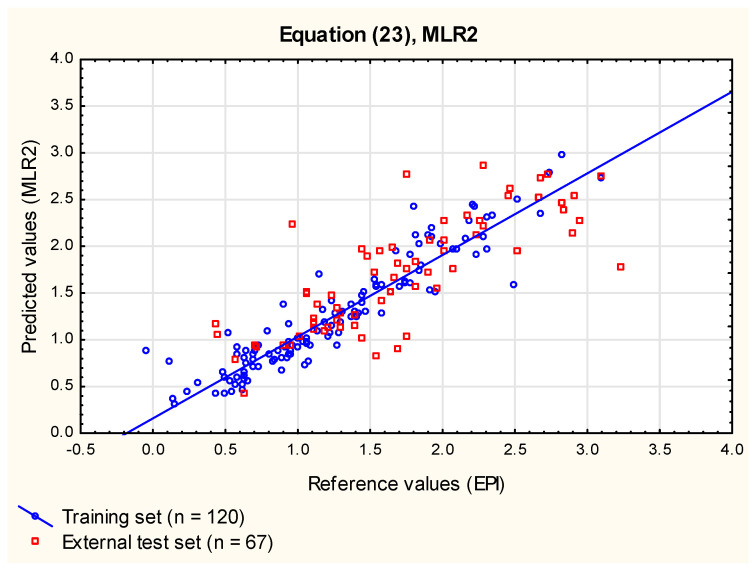
**MLR2** model, Equation (23)—predicted vs. reference log ***BCF*** values.

The results of log ***BCF***_EPI_ modeling using a single chromatographic descriptor (log ***k_w_***^IAM^) obtained in this study (Equation (22), **MLR1**) are similar to those reported by Tsopelas [4] (R^2^ = 0.74, *n* = 77). Log ***k_w_***^IAM^ accounted for ca. 74% of variability of log ***BCF_vivo_*** data (which proves the importance of the chromatographic parameter), but it was hoped that the model can be improved by incorporating some additional independent variables expected to influence the ability of compounds to be absorbed by aquatic animals from the surrounding water via the respiratory route and skin. It is likely that, similarly to pharmacokinetic processes of compound absorption and distribution in humans, the key features responsible for the ability of molecules to bioconcentrate in aquatic organisms are their lipophilicity (which, indeed, is the main parameter in the majority of ***BCF*** in silico models), ability to form hydrogen bonds and molecule flexibility and size. Apart from log ***k_w_***^IAM^, which is strongly related to solutes’ lipophilicity, several molecular descriptors calculated using SwissADME software were investigated. The improved Equation (23) (**MLR2**, Figure 2) was generated using forward stepwise regression:

log ***BCF_EPI_*** = 0.27 (± 0.06) + 0.71 (± 0.03) log ***k_w_***^IAM^ − 0.0043 (± 0.0009) ***TPSA***+ 0.24 (± 0.06) ***F_Csp3_*** − 0.089 (± 0.036) ***HD*** (23)

(*n* = 120, R^2^ = 0.87, R^2^_adj_ = 0.87, RMSECV = 0.25, RMSEP_ext_ = 0.42, RMSEP_vivo_ = 0.27, R^2^_vivo_ = 0.83, F = 198.4, *p* < 0.01).

The additional independent variables incorporated into Equation (23) (**MLR2**) were statistically significant and accounted for ca. 7% of total variability. They were introduced in the following order: ***TPSA***, ***F_Csp3_*** and ***HD***, which confirmed the relationship between ***TPSA*** and the phenomenon of bioconcentration reported earlier by Tsopelas [4] (who also demonstrated the contribution of a biodegradation estimate, ***BioWin5***, calculated using EPISuite^TM^ software). Polar surface area is an important parameter that defines the polar part of a molecule. It is strongly related to the passive transport of molecules through membranes, and it is known to influence the ADME processes in humans (e.g., the blood and brain barrier permeability, transdermal or intestinal absorption [62,63,64]). Other ***BCF*** predictors incorporated in Equation (23) (**MLR2**, Figure 2) are the fraction of sp^3^ carbons ***F_Csp3_*** (which, in simple terms, can be considered a measure of molecule’s flexibility and is positively correlated with log ***BCF***) and the count of H-bond donors ***HD***. The coefficients for both ***HD*** and ***PSA*** in Equation (MLR2) are negative—high polar surface area and the molecule’s strong tendency to form hydrogen bonds reduce its uptake by aquatic organisms.

**Figure 3:**
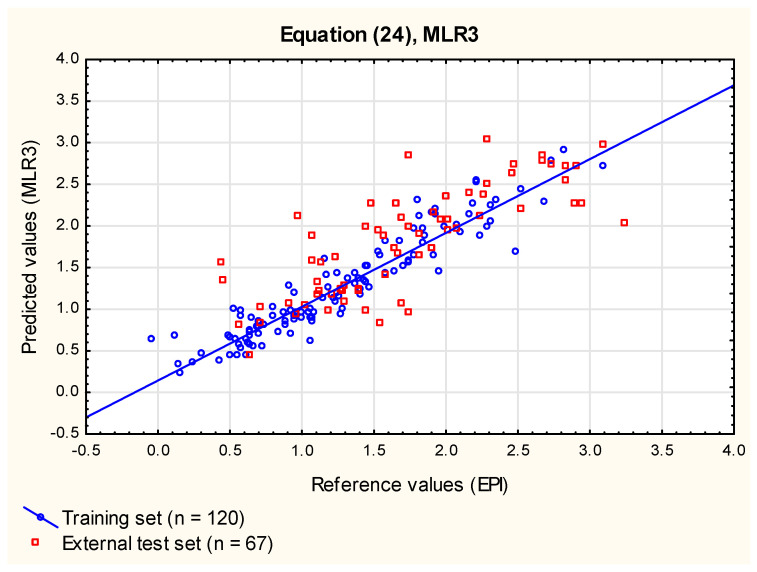
**MLR3** model, Equation (24)—predicted vs. reference log ***BCF*** values.

Further attempts to improve the MLR models by incorporating other parameters expected to influence the compounds’ ability to bioconcentrate were not very successful—Equation (24) (**MLR3**, Figure 3), obtained using six variables selected by forward stepwise regression, had slightly better parameters of cross-validation than the model **MLR2** (Equation (23)), but this gain didnot justify the risk of over-fitting related to incorporation of two more parameters (***FRB*** and ***DipH***) that, although both statistically significant, accounted together for only slightly over 1% of total variability. The ability of Equation (24) to predict log ***BCF*** for new cases (the external test set) and the relationship between log ***BCF*** values predicted using this model and the experimental values were comparable to those reported for Equation (23) (**MLR2**).

log ***BCF_EPI_*** = 0.14 (± 0.06) + 0.74 (± 0.03) log ***k_w_***^IAM^ − 0.0037 (± 0.0011) ***TPSA*** + 0.35 (± 0.07) ***F_Csp3_*** − 0.16 (± 0.04) ***HD*** − 0.026 (± 0.011) ***FRB*** + 0.29 (± 0.08) ***DipH*** (24)

(*n* = 120, R^2^ = 0.89, R^2^_adj_ = 0.88, RMSECV = 0.17, RMSEP_ext_ = 0.45, R^2^_vivo_ = 0.83, RMSEP_vivo_ = 0.27, F = 147.3, *p* < 0.01). 

### 3.2. Partial Least Square (PLS) Models

In this study, the following PLS models were investigated (details to be found in Supplementary Materials):

- Models **PLS1** based on 16 independent variables—including those involved in MLR analysis and some other descriptors that were not included in MLR to avoid colinearity problems;
- Model **PLS2** based on a reduced set of independent variables.

**Figure 4:**
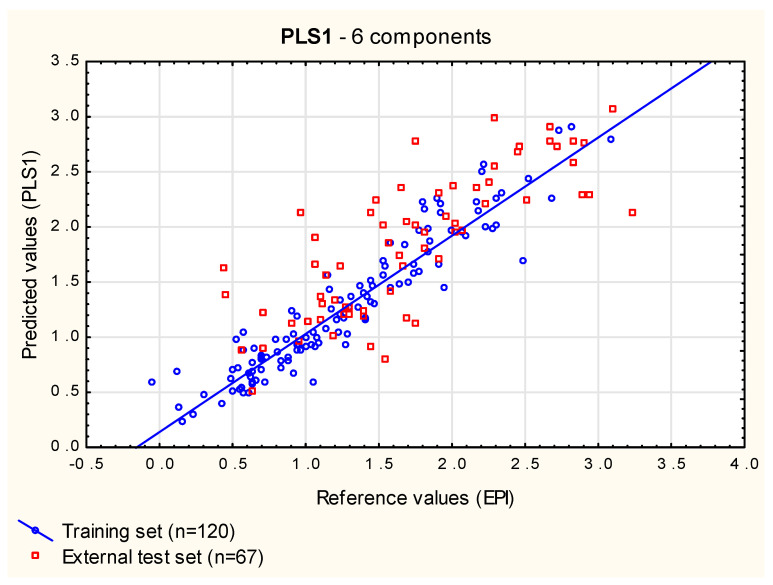
**PLS1** model (six components)—predicted vs. reference log ***BCF*** values.

**PLS1** models based on the set of 16 independent variables and involving between 4 and 12 components were compared using RMSEP_ext_, RMSEP_vivo_ and RMSECV values (Supplementary Materials). At a later step, multiple linear forward stepwise regression was also performed on the X-scores of all the possible 16 PLS components. Using these two approaches, it was established that the optimum number of components is six (Figure 4)—it led to a model that fitted the training dataset reasonably well, the model’s predictive potential was satisfying (i.e., the model was neither over-fitted or under-fitted) and all six PLS components selected by MLR were statistically significant. 

**Figure 5:**
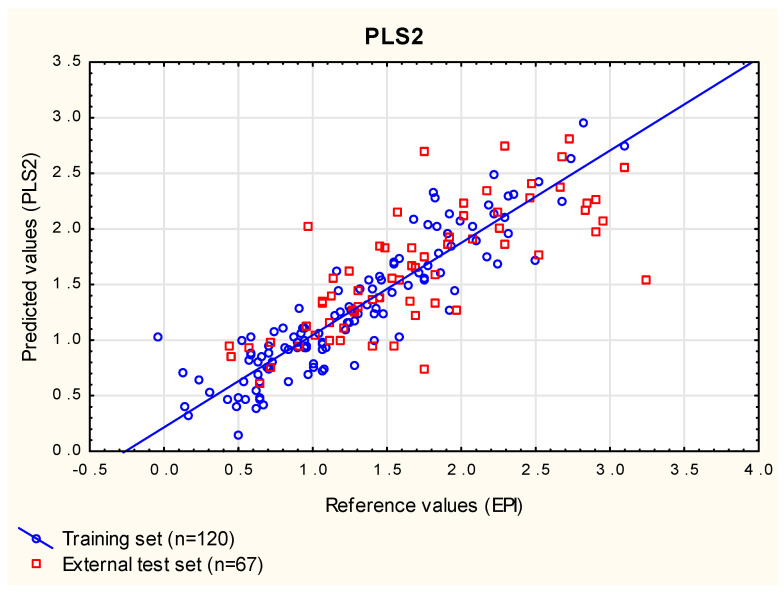
**PLS2** model—predicted vs. reference log ***BCF*** values.

The importance of descriptors used in PLS models can be evaluated manually based on their variable importance in the projection (VIP) values calculated for the particular number of components (descriptors with VIP < 1 in a PLS model are excluded from the next one) [65] (Table 2). This procedure was applied to **PLS1**, and it was established that only two variables, log ***k_w_***^IAM^ and ***MR*** (a descriptor connected with polarizability of molecules, not selected in MLR) had a strong influence on log ***BCF*** (model **PLS2**, Figure 5). Surprisingly, the descriptors selected by stepwise multiple regression (apart from log ***k_w_***^IAM^, which is of utmost importance in all the models developed in this study) were of lesser importance in the PLS regression. Model **PLS2**, however, seemed excessively simplified, and its performance, evaluated using RMSECV, RMSE_ext_ and RMSE_vivo_, was slightly worse than that of **PLS1** (Table 3).

### 3.3. Artificial Neural Networks

Artificial neural networks are widely used to predict drugs’ bioavailability [66] or properties such as affinity for phospholipids using IAM chromatography and calculated descriptors [67]. The great advantages of neural networks compared to MLR are the possibility of utilizing both linear and non-linear relationships between input data and a predicted parameter and the ability of ANNs to learn these relationships directly from the data being modeled.

**Figure 6:**
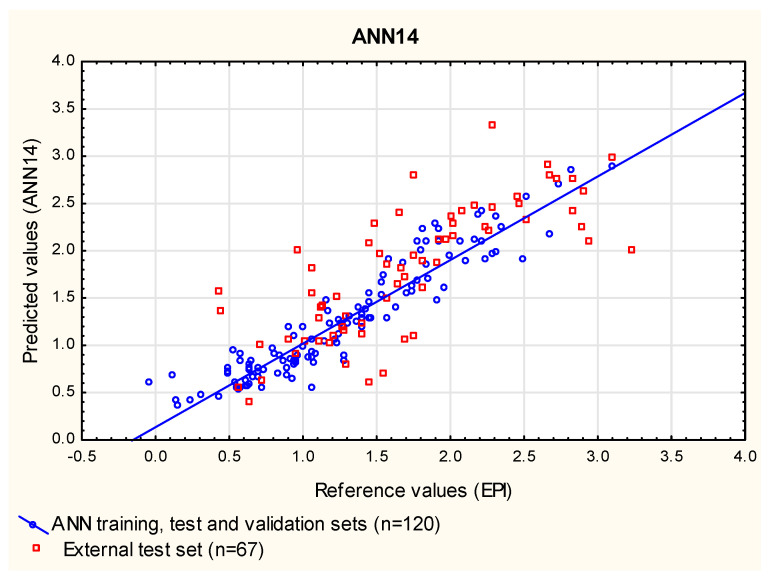
**ANN14** model—predicted vs. reference log ***BCF*** values.

**Figure 7:**
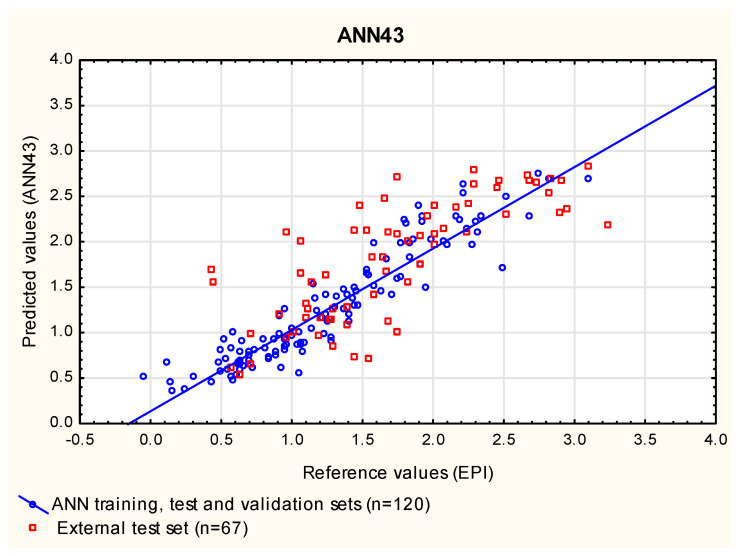
**ANN43** model—predicted vs. reference log ***BCF*** values.

**Figure 8:**
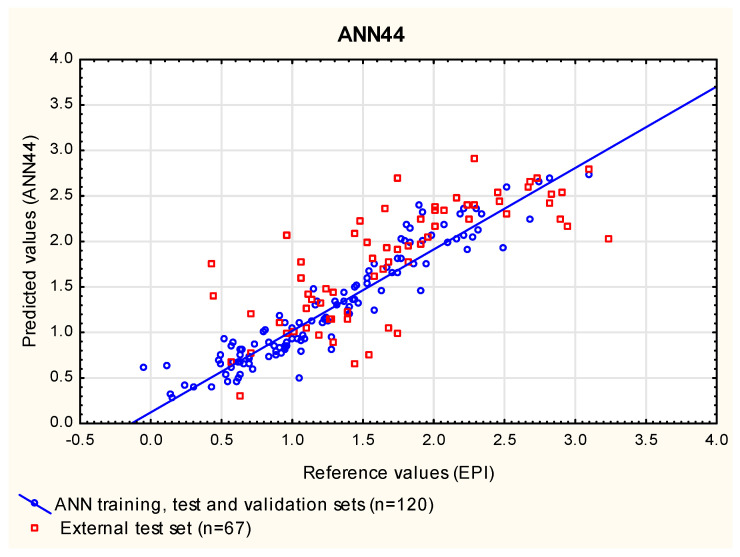
**ANN44** model—predicted vs. reference log ***BCF*** values.

In this study, the ANN models were built for the same group of compounds (***1*** to ***120***) that was used as the training set in the MLR and PLS analyses. This group of compounds was randomly assigned to three subgroups: train (70%), test (15%) and validation (15%)—the latter two groups were needed to optimize the ANNs as they were being created. Similarly to the MLR and PLS analyses presented in this study, the compounds ***121*** to ***240*** were used as an additional, external test set. At this point, 1000 networks were generated, and 50 with the smallest error were retained for further examination in search of those that give the results in the closest agreement with the reference data (log ***BCF_EPI_***) for compounds ***121*** to ***187*** (RMSEP_ext_) and with the experimental data (log ***BCF_vivo_***) for a subgroup of 40 cases, whose experimental log ***BCF*** values were available (R^2^_vivo_, RMSEP_vivo_). The selection of the best exemplary networks generated in this study (**ANN14**, **ANN43** and **ANN44**, Figure 6, Figure 7 and Figure 8) was based on their ability to predict new cases (RMSEP_ext_) and to obtain the results in the closest possible agreement with the experimental data (R^2^_vivo_, RMSEP_vivo_) rather than on their ability to fit the training data (Supplementary Materials).

ANNs make it possible to process a large number of descriptors that can be easily obtained using readily available software. The selection of ANN input data is an important step because, if the number of parameters is excessive considering the number of cases, models are over-fitted. The importance of independent variables can be evaluated using a tool known as global sensitivity analysis (GSA), which rates the importance of the models’ input variable by computing sums of squared residuals for the model when the respective predictor is eliminated compared to the full model. When an input variable scores 1 or less than 1 in GSA, it means that this particular network is likely to perform better without this variable; however, in the networks generated in this study, the majority of GSA scores were at least slightly above this threshold.

Log ***k_w_***^IAM^ is an important predictor accounting for 80% of log ***BCF*** variability. It encodes the molecule’s properties responsible for its ability to cross biological membranes—lipophilicity and size (molecular weight, heavy atom count), Table 4—and, when additional descriptors are incorporated, it leads to efficient ***BCF*** models. In this study, the models were generated using log ***k_w_***^IAM^ values obtained directly for aqueous mobile phases. Using the external test group of solutes, it was demonstrated, however, that log ***k_w_***^IAM^ values obtained by extrapolation of log ***k***^IAM^ values to zero concentration of organic modifiers in the mobile phase were sufficient to give reasonable predictions—although, since log ***k_w_***^IAM^ is the most important descriptor in all the models, imperfections of this variable in the external test dataset always had some influence on the RMSEP_ext_ values.

**Figure 9:**
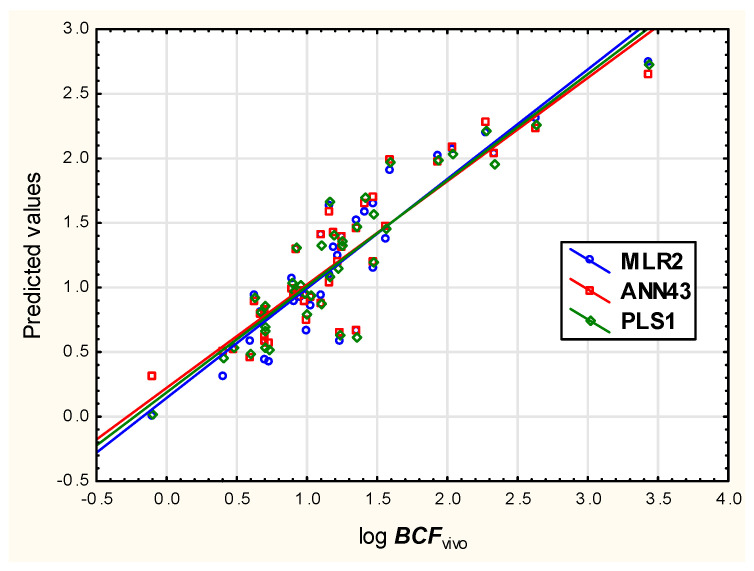
Predicted vs. experimental log ***BCF*** values for models MLR2, PLS1 and ANN43.

Models **MLR2**, **PLS1** and **ANN43** were finally compared (Figure 9) by plotting the predicted log ***BCF*** values against the experimental ones (log ***BCF_vivo_***), and it was confirmed that their ability to model the experimental log ***BCF*** data was similar.

## 4. Conclusions

The ability of compounds to bioconcentrate in aquatic organisms is strongly related to their affinity for phosphatydylocholine-based immobilized artificial membranes (IAM), and other physico-chemical parameters of a molecule are less important in this process. QSAR models of log ***BCF*** involving the IAM chromatographic retention factor and other descriptors were built using multiple linear regression, partial lest square regression and artificial neural networks. The MLR approach is a powerful technique with the great advantage of simplicity—models generated using this technique usually involve a relatively small number of independent variables (parameters), whose physical meaning and contribution towards an dependent variable can be easily understood. In this study, the selected MLR, PLS and ANN models gave fairly comparable results in terms of their ability to predict new cases (log ***BCF_ext_***), and the results obtained using these models were in similar agreement with experimental data (log ***BCF_vivo_***) (surprisingly, simple MLR equations based on a relatively small number of independent variables seemed to perform slightly better than more complex ANN or PLS models). Generally speaking, PLS regression deals with the colinearity of independent variables, and the ANN approach is especially useful in the case of non-linear relationships, but, in this study, linear equations (especially Equation (23), **MLR2**) gave satisfying prediction results, and they were more intuitive. All the models reported above can be easily applied during the early steps of the drug discovery process concurrently with IAM chromatographic pharmacokinetic studies and, as described earlier, in the studies of compounds’ mobility in the soil–water compartment [6]. In lieu of log***k_w_***^IAM^ obtained directly using aqueous mobile phases, extrapolated values can be used, although, in such situations, the quality of ***BCF*** predictions is slightly impaired. The models proposed in this study are applicable to compounds over a relatively wide range of lipophilicity, with the exception of very lipophilic molecules (log ***K_ow_*** > *ca*. 7), whose retention times on the IAM chromatographic support are very long and log ***k_w_***^IAM^ cannot be conveniently measured. This limitation of the applicability domain of the models presented in this study, however, is not a major drawback—very lipophilic compounds, as demonstrated by some authors, do not bioconcentrate or bioaccumulate easily [16,27,28,34], which is either a direct result of their hydrophobicity or, indirectly, an effect of the larger molecular size of highly lipophilic molecules [32]. Above a certain lipophilicity threshold (log ***K_ow_*** > ca. 7), the bioconcentration factor becomes inversely proportional to lipophilicity and decreases rapidly. On the other hand, a large proportion of compounds released to the environment by agriculture or the pharmaceutical industry (e.g., pesticides or drugs) meets the criteria of optimum intestinal, transdermal or lung absorption [19,68,69,70,71,72]. Such compounds are usually moderately lipophilic (log ***K_ow_*** rarely higher than 7, in the majority of cases, between 0 and 5), so quantitative studies of their bioconcentration using the models discussed above are feasible.

## Figures and Tables

**Table 1:** log ***BCF*** vs. log ***K_ow_*** according to Meylan (^a^) and US EPA (^b^) models.

log *K_ow_*	Non-Ionic		log *K_ow_*	Ionic	
	Meylan	US EPA		Meylan ^a^	US EPA ^b^
below 1	0.50	0.50	below 5	0.50	
1 to 7	0.77 log ***K_ow_***− 0.70 + Σ***F_i_***	0.6598 log ***K_ow_***− 0.333 + Σ***F_i_***	5 to 6	0.75	
7 to 10.5	−1.37 log ***K_ow_***+ 14.4 + Σ***F_i_***	−0.49 log ***K_ow_***+ 7.554 + Σ***F_i_***	6 to 7 ^a^ or 8 ^b^	1.75	
			7 ^a^ or 8 ^b^ to 9	1.00	
above 10.5	0.50		above 9	0.50	

where Σ***F_i_***—sum of correction factors.

**Table 2:** VIP values for independent variables, model **PLS1**.

Variable	VIP	Importance
log ***k_w_***^IAM^	2.53	1
***MR***	1.08	2
***#HvAt***	0.97	3
***M_w_***	0.97	4
***HD***	0.92	5
***DipPCh***	0.88	6
***E_t_***	0.84	7
***FRB***	0.84	8
***DipS***	0.83	9
***TPSA***	0.76	10
***#ArHvAt***	0.72	11
***DipH***	0.71	12
***E_HOMO_***	0.69	13
***HA***	0.64	14
***E_LUMO_***	0.48	15
***F_Csp3_***	0.32	16

**Table 3:** Summary of MLR, PLS and ANN models developed in this study.

	MLR1	MLR2	MLR3	PLS1	PLS2	ANN14	ANN43	ANN44
RMSECV	0.30	0.25	0.17	0.26	0.29	-	-	-
RMSEP_ext_	0.35	0.42	0.45	0.45	0.46	0.47	0.47	0.47
RMSEP_vivo_	0.35	0.27	0.27	0.27	0.31	0.28	0.28	0.30
R^2^_vivo_	0.74	0.83	0.83	0.83	0.77	0.81	0.82	0.79

**Table 4:** Correlations between descriptors analyzed in this study.

	log *k_w_*^IAM^	*M_W_*	*#HAt*	*#ArHAt*	*F_Csp3_*	*FRB*	*HA*	*HD*	*MR*	*TPSA*	*E_t_*	*E_HOMO_*	*E_LUMO_*	*DipPCh*	*DipH*	*DipS*	log *K_ow_*
log ***k_w_***^IAM^	1.00	0.51	0.51	0.39	−0.07	0.34	0.09	−0.15	0.57	−0.08	−0.44	0.38	−0.17	−0.09	0.05	−0.04	0.84
***M_w_***	**0.51**	1.00	0.98	0.50	0.07	0.62	0.78	0.38	0.98	0.64	−0.86	0.49	−0.42	0.45	0.52	0.49	0.32
***#HAt***	**0.51**	0.98	1.00	0.52	0.06	0.63	0.78	0.37	0.99	0.64	−0.87	0.53	−0.39	0.46	0.52	0.49	0.32
***#ArHAt***	0.39	0.50	0.52	1.00	−0.58	0.16	0.27	0.02	0.53	0.17	−0.44	0.56	−0.52	0.13	0.40	0.16	0.29
***F_Csp3_***	−0.07	0.07	0.06	−0.58	1.00	0.27	0.09	0.06	0.08	0.00	−0.01	−0.29	0.59	−0.01	−0.07	−0.01	−0.04
***FRB***	0.34	0.62	0.63	0.16	0.27	1.00	0.59	0.31	0.63	0.45	−0.52	0.31	−0.06	0.26	0.33	0.31	0.25
***HA***	0.09	0.78	0.78	0.27	0.09	0.59	1.00	0.53	0.70	0.86	−0.75	0.25	−0.39	0.61	0.54	0.61	−0.11
***HD***	−0.15	0.38	0.37	0.02	0.06	0.31	0.53	1.00	0.33	0.67	−0.35	0.26	−0.14	0.26	0.52	0.28	−0.27
***MR***	**0.57**	0.98	0.99	0.53	0.08	0.63	0.70	0.33	1.00	0.57	−0.84	0.56	−0.36	0.41	0.51	0.44	0.38
***TPSA***	−0.08	0.64	0.64	0.17	0.00	0.45	0.86	0.67	0.57	1.00	−0.63	0.21	−0.43	0.67	0.55	0.68	−0.28
***E_t_***	−0.44	−0.86	−0.87	−0.44	−0.01	−0.52	−0.75	−0.35	−0.84	−0.63	1.00	−0.43	0.42	−0.48	−0.42	−0.49	−0.25
***E_HOMO_***	0.38	0.49	0.53	0.56	−0.29	0.31	0.25	0.26	0.56	0.21	−0.43	1.00	−0.23	0.13	0.44	0.19	0.26
***E_LUMO_***	−0.17	−0.42	−0.39	−0.52	0.59	−0.06	−0.39	−0.14	−0.36	−0.43	0.42	−0.23	1.00	−0.39	−0.27	−0.38	−0.05
***DipPCh***	−0.09	0.45	0.46	0.13	−0.01	0.26	0.61	0.26	0.41	0.67	−0.48	0.13	−0.39	1.00	0.33	0.97	−0.28
***DipH***	0.05	0.52	0.52	0.40	−0.07	0.33	0.54	0.52	0.51	0.55	−0.42	0.44	−0.27	0.33	1.00	0.44	−0.11
***DipS***	−0.04	0.49	0.49	0.16	−0.01	0.31	0.61	0.28	0.44	0.68	−0.49	0.19	−0.38	0.97	0.44	1.00	−0.25
log ***K_ow_***	**0.84**	0.32	0.32	0.29	−0.04	0.25	−0.11	−0.27	0.38	−0.28	−0.25	0.26	−0.05	−0.28	−0.11	−0.25	1.00

## Data Availability

The data presented in this study are available in this manuscript.

## Supplementary Materials

The supporting information can be downloaded at: https://www.mdpi.com/article/10.3390/membranes12111130/s1 as an Excel file: Supplementary Materials.xlsx.
